# Spontaneous Suppressors against Debilitating Transmembrane Mutants of *Ca*Mdr1 Disclose Novel Interdomain Communication via Signature Motifs of the Major Facilitator Superfamily

**DOI:** 10.3390/jof8050538

**Published:** 2022-05-22

**Authors:** Suman Sharma, Atanu Banerjee, Alexis Moreno, Archana Kumari Redhu, Pierre Falson, Rajendra Prasad

**Affiliations:** 1 Amity Institute of Biotechnology, Amity University Haryana, Gurugram 122413, India; sssumansharma21@gmail.com (S.S.); abanerjee1@ggn.amity.edu (A.B.); 2 Drug Resistance & Membrane Proteins Team, Molecular Microbiology and Structural Biochemistry Laboratory, Institut de Biologie et Chimie des Protéines, CNRS-Lyon 1 University Research lab n° 5086, 69367 Lyon, France; amoreno@calixar.com; 3 ACTREC, Tata Memorial Centre, Navi Mumbai 410210, India; archana.redhu@yahoo.com; 4 Amity Institute of Integrative Sciences and Health, Amity University Haryana, Gurugram 122413, India

**Keywords:** *Candida albicans*, antifungal resistance, MFS transporter, *Ca*Mdr1, drug:H^+^ antiporter 1, interdomain crosstalk, Signature motifs

## Abstract

The Major Facilitator Superfamily (MFS) drug:H^+^ antiporter *Ca*Mdr1, from *Candida albicans*, is responsible for the efflux of structurally diverse antifungals. MFS members share a common fold of 12–14 transmembrane helices (TMHs) forming two N- and C-domains. Each domain is arranged in a pseudo-symmetric fold of two tandems of 3-TMHs that alternatively expose the drug-binding site towards the inside or the outside of the yeast to promote drug binding and release. MFS proteins show great diversity in primary structure and few conserved signature motifs, each thought to have a common function in the superfamily, although not yet clearly established. Here, we provide new information on these motifs by having screened a library of 64 drug transport-deficient mutants and their corresponding suppressors spontaneously addressing the deficiency. We found that five strains recovered the drug-resistance capacity by expressing *Ca*Mdr1 with a secondary mutation. The pairs of debilitating/rescuing residues are distributed either in the same TMH (T127A_TMH1_- > G140D_TMH1_) or 3-TMHs repeat (F216A_TMH4_- > G260A_TMH5_), at the hinge of 3-TMHs repeats tandems (R184A_TMH3_- > D235H_TMH4_, L480A_TMH10_- > A435T_TMH9_), and finally between the N- and C-domains (G230A_TMH4_- > P528H_TMH12_). Remarkably, most of these mutants belong to the different signature motifs, highlighting a mechanistic role and interplay thought to be conserved among MFS proteins. Results also point to the specific role of TMH11 in the interplay between the N- and C-domains in the inward- to outward-open conformational transition.

## 1. Introduction

*C. albicans* is a commensal that can become pathogenic and cause serious infections, particularly under compromised immunity in the human host. Among the various strategies adopted by the yeast to resist the antifungal onslaught, elevated drug efflux significantly contributes to an expeditious advent of antifungal resistance [1]. This reduced intracellular accumulation of drugs in *Candida* is predominantly accredited to *Ca*Cdr1 and *Ca*Cdr2, belonging to the ATP-binding cassette (ABC) transporter proteins, and the MFS protein *Ca*Mdr1 [2,3,4,5,6].

MFS members are extensively distributed across many domains of life [7,8], forming the broadest and most renowned superfamily of secondary transporters that comprises 105 families (http://tcdb.org/superfamily.php; accessed on 14 November 2020; [9]). MFS members operate as uniporters, symporters, and antiporters. They are unique in exhibiting a wide spectrum of substrates [10]. Symporters and antiporters take the advantage of the electrochemical potential of co-transported solute or ion, whereas uniporters mediate the facilitated diffusion of a single type of substrate along their concentration gradient [11]. Most of the MFS proteins share a common scaffold for all members of the family, made of 12–14 TMHs [12]. The genome of *C. albicans* features 95 MFS proteins divided into 17 families [13]. Among them, the Drug:H^+^ Antiporter 1 (DHA1) family (which contains 22 transporters, including *Ca*Mdr1) utilizes an electrochemical gradient of protons to facilitate the transport of cargo against its concentration gradient across the membrane [14]. Among the *C. albicans* MFS proteins, only *Ca*Mdr1 has been linked to a resistant phenotype towards azole antifungals, as well as several unrelated drugs such as 4-nitroquinoline–N-oxide, cycloheximide, benomyl, methotrexate, and cerulenin [14,15,16,17].

The majority of the structural information has come from prokaryotic MFS transporters and, to a lesser extent, from eukaryotic homologs [18,19,20,21,22,23,24,25,26,27]. 12-TMH MFS proteins are made of two N- and C- 6-TMH subdomains that are organized as two inverted pairs of 3-TMH bundles [25,28,29]. MFS members display poor primary structure identity but share a few conserved signature motifs thought to play similar key roles [30,31]. Motif A (*GxLaD^180^rxGrkx_3_I,* referring to the *Ca*Mdr1 sequence numbering) is located within the cytoplasmic loop between TMH2 and TMH3. It was considered to be involved in the inward/outward conformational change [31], and was later found to be stabilizing the outward-facing conformation of YajR [21] by salt-bridges either in the A motif or with adjacent regions [32,33]. Motif B (*Ix_3_R^215^x_2_qGxga_2_*) is located in the external leaflet of TMH4. It contains an arginine residue thought to be involved in proton transfer, which we also confirmed for *Ca*Mdr1 [34]. Motif C (*gx_3_G^260^Px_2_G_2_xI*) is positioned in the external leaflet of TMH5. It displays two Gx_3_G motifs, known to stabilize helix–helix association in membrane proteins through interaction with bulky side-chain residues [35]. Mutation of these glycine residues in TetA [36] and *Ca*Mdr1 [37] is indeed critical. Motif D (*lgx_5_P^139^vxP*) in TMH1 and motif G (*Gx_3_GPL^512^*) in TMH11 are exclusive to the 12- (motif D) and 12–14-TMH (motif G) families, respectively [30]. Thus far, both motifs have been poorly investigated, but alanine scanning of the corresponding regions of *Ca*Mdr1 (*MGSAVYTP^139^GIE* and *IASVFPL^512^*) showed that residues M132, Y137, V506, A508, P512, and L513 are indeed structurally or functionally critical [38].

The *Escherichia coli* lactose/H^+^ symporter LacY has been the most extensively studied among all the MFS members. Its X-ray structures in inward-open and ligand-bound occluded conformations provided a prototype for understanding the transport mechanism [39,40,41,42]. Several elegant structural studies in the last decade allowed the visualization of multiple substrate-bound transporter conformations, from which a general alternative access mechanism of transport has been deduced. Mechanistically, each protein has a single substrate-binding cavity in the center of the membrane domain. The switch between the inward-open and outward-open conformations at the N- and C-domain interface exposes this cavity to either side of the membrane. N- and C-moieties contribute asymmetrically to form the substrate-binding pocket in symporters and facilitators, whereas they contribute equally in antiporters [25,26]. The presence of aromatic residues in the cavity prevents the exposure of the substrate to the cytosolic or extracellular sides [25]. Detailed biochemical, biophysical, and structural investigations of the MFS antiporters MdfA, EmrD, YajR, and SotB from *E. coli* and LmrP from *Lactococcus lactis* revealed that the substrate–H^+^ coupling mechanism involves the sequential binding and release of substrate and proton. Both halves of the protein move correlatively similar to a rocker switch, arbitrated by salt bridge formation and breakage during the transport cycle [21,43,44,45,46,47]. Further studies suggest that although proton translocation and substrate transport occur in distinct sites, they always compete for protein binding. Consequently, protonation leads to conformation changes of the protein that facilitate substrate uptake from the intracellular side (inward-open conformation), whereas deprotonation destabilizes the substrate-bound state of the protein and eventually leads to substrate release on the extracellular side (outward open conformation) [19,26,48,49,50,51].

Our groups have been focusing on the functional aspects of *Ca*Mdr1, mainly by subjecting it to site-directed mutagenesis and homology modeling [34,37,38,52,53]. These studies firstly highlighted the role of a central cytoplasmic loop (CCL or ICL_3_) in establishing contact between the protein and the plasma membrane [52]. Then, site-directed mutagenesis guided by prediction of critical residues based on information theoretic measures allowed us to identify several functionally relevant residues [54]. Finally, the systematic replacement by alanine of the 252 residues forming the membrane domain of *Ca*Mdr1 revealed 84 residues critical for drug efflux, which we categorize depending on their type and impact on *Expression* (protein expression and plasma membrane localization), *Structure* (typically glycine, proline, and alanine residues), interaction with *Lipids* (i.e., facing the membrane), *Mechanism* (buried residues not facing the drug-binding cavity), substrate *Binding* (buried residues facing the drug-binding cavity), and *Polyspecificity* (same as *Binding* but displaying substrate selectivity). Notably, the spatial organization of residues belonging to the two last groups draw structural features of the drug polyspecificity characterizing such proteins [38].

These studies provide a fair understanding of drug–protein interaction, but lack information about the dynamics of the mechanism and interaction between critical residues. To explore these aspects, we took advantage of our in-house library of critical mutants, which we subjected to the suppressor genetics strategy. This led to 16 strains recovering resistance to antifungals from initial transport-sensitive mutants belonging to the *Structure/Lipids*, *Mechanism*, *Binding*, and *Polyspecificity* groups. Among them, only strains expressing mutants from the two first groups led to stable and intragenic secondary mutations which, strikingly, target the conserved MFS motifs, delivering new information on short- and long-range interactions of the antiporter and role of these motifs.

## 2. Materials and Methods

### 2.1. Materials

#### 2.1.1. Reagents

All routine chemicals used in the study were acquired from Himedia, Merck, or SRL Pvt. Ltd. Mumbai, India. Drugs such as cycloheximide (CHX), 4-nitroquinoline 1-oxide (NQO), fluconazole (FLC), anisomycin (ANI), cerulenin (CER), and fluorescent dye Nile red (NR) were procured from Sigma-Aldrich Co., St. Louis, MO, USA. Reagents such as ammonium acetate, Polyethylene Glycol (PEG), Lithium Acetate (LiAc), sodium chloride (NaCl), Tris-HCl, EDTA, and dimethyl sulfoxide (DMSO) were also purchased from Sigma-Aldrich Sigma-Aldrich Co., St. Louis, MO, USA. Oligonucleotides were obtained from Sigma-Aldrich, Bangalore, India and are listed in the Table S1. 

#### 2.1.2. Strains and Culture Conditions

All *Saccharomyces cerevisiæ* strains were grown either in YEPD (1% Yeast extract, 2% Peptone, and 2% Dextrose) broth or on solid YEPD plates with 2% agar with or without drugs as per the experimental requirements. The Dh5α strain of *E. coli* was used to maintain all plasmids and was grown in Luria Bertani media with 100 mg/L ampicillin (Amresco, Solon, Cleveland, OH, USA). Both growth media (YEPD and LB media) and agar were purchased from HiMedia Laboratories, Mumbai, India. To select yeast transformants, we used a synthetic defined medium without uracil (SD-Ura^-^) plates that was composed of 0.67% Yeast nitrogen base without amino acids (Difco, Becton, Dickinson and Company, MD, USA), 0.2% Ura^-^ dropout mix (Sigma-Aldrich Co., St. Louis, MO, USA), and 2% glucose (Merck, Darmstadt, Germany), along with 2.5% (*w/v*) agar from Hi Media laboratories, Mumbai, India. All yeast and bacterial strain stocks were prepared using 15% glycerol and stored at −80 °C. Table S2 lists all the strains used in this study.

### 2.2. Methods

#### 2.2.1. Generation and Sequence Analysis of Suppressor Mutants

Cultures of yeast grown overnight expressing the *Ca*Mdr1-GFP mutant variants were washed with sterile 0.9% saline (0.9% sodium chloride) solution. The cells were then homogenously mixed with 25 mL molten YEPD agar to accomplish a final OD_600nm_ of 10^5^ cells/mL and were poured into Petri plates. The filter discs were positioned on the plates once the medium had solidified, and the desired toxic concentration of drugs was deposited on the discs using a pipette. Afterwards, yeast cells were allowed to grow under the selective pressure of its drug substrates for 6–7 days at 30 °C. The plates were observed regularly, and the colonies that appeared within the inhibitory zone were picked up and subsequently validated by passage on drug plates. Further, genomic DNA was extracted from the validated colonies using TENTS buffer as described previously [55], and the *CaMDR1* gene was amplified by performing PCR using Phusion polymerase from New England Biolabs, Ipswich, MA, USA and *CaMDR1* full gene primers listed in Table S1, manufactured by Sigma-Aldrich, Bangalore, India. To detect base alterations that resulted in amino acid substitution, PCR amplicons were sequenced using overlapping primers across the whole *CaMDR1* ORF. To avoid errors, the sequencing was done at least twice. Align Me software was used for sequence alignment to identify nucleotide substitutions in the ORFs that led to any change in the amino acid sequence of *Ca*Mdr1 in the resistant colonies.

#### 2.2.2. Site-Directed Mutagenesis and Yeast Transformation

To perform site-directed mutagenesis, a Quick-Change kit was used as per the manufacturer’s protocol (Agilent Technologies, Santa Clara, CA, USA). A full plasmid with the *CaMDR1* gene was amplified by using pre-designed primers harboring the desired mutation. The oligonucleotides used to insert the mutation(s) are listed in Table S1. The PCR product was then digested with *Dpn*1 (New England Biolabs, Ipswich, MA, USA) to destroy the wild type template, and the digested product was used to transform Dh5α cells. DNA sequencing was performed to verify the colonies carrying desired mutations. Following confirmation, the plasmids were digested with *Xba*I (New England Biolabs, Ipswich, MA, USA) restriction enzyme to liberate the linearized plasmid. This linearized plasmid (pSKPPUS-*CaMDR1*-GFP) was then directly used to transform into *S. cerevisiæ* AD1-8u^-^ cells using the well-established LiAc-based technique [56]. Transformants were selected on SD-Ura^-^ agar plates [37]. Strains generated and used in the present study are listed in Table S2.

#### 2.2.3. Confocal Microscopy

The exponential phase cells of AD1-8u-expressing GFP-tagged protein variants at their C-terminal were first pelleted and then rinsed with 1× Phosphate Saline Buffer (PBS). The cells were then examined under a Nikon A1 confocal laser microscope with a 60× oil immersion objective lens.

#### 2.2.4. Spot Dilution Growth Assay

For the serial dilution spot assay, yeast cells grown overnight were suspended in 0.9% saline (0.9% sodium chloride) solution at a final OD_600nm_ of 10^6^ cells/mL and were then serially diluted five times. A 4 µL aliquot from each dilution was put on YEPD agar plates either with or without the xenobiotics. Plates were kept at 30 °C for 48 h [57] and final images were taken using the BIO-RAD ChemiDoc^TM^ XRS+ system.

#### 2.2.5. MIC Assay

Minimum inhibitory concentrations were determined using the Broth microdilution assay as described previously [58].

#### 2.2.6. Nile Red Accumulation Assay

Cells were grown until the exponential phase from overnight culture in YEPD broth. Then, 0.25 OD_600nm_ cells were harvested and washed with 1x PBS and resuspended in a dilution medium (containing 1/3 YEPD and 2/3 water) as described previously [59]. Then, Nile red was added at a final concentration of 7 μM to the cells, which were then incubated at 30 °C with 200 rpm for 30 mins. Afterwards, cells were collected by centrifugation and washed thrice with 1× PBS before analysis on a BD FACSLyric^TM^ flow cytometer. Ten thousand cells were used for each strain to detect the geomean fluorescence intensity within the cells. The data were analyzed with BD FACSuite software. Finally, bar graphs were prepared for fluorescence intensity values by considering accumulation as 100% in the control (AD1-8u^−^) strain. 

#### 2.2.7. WebLogo Generation

Sequences were downloaded from the PFAM webserver, using 192 “seed” sequences from the MFS_DHA1 subfamily (PF07690). Sequence alignment was performed using the Jalview 2.11.1.4. Weblogo was generated by using the web-based application WebLogo 3, using Chemistry color coding (polar—green; neutral—purple; basic—blue; acidic—red; hydrophobic—blue). At the bottom of the Y-axis, numbers denote the amino acid residue number of *Ca*Mdr1 (123–512).

#### 2.2.8. Generation of 3D Homology Model of *Ca*Mdr1

A 3D model of the outward-facing conformation of *Ca*Mdr1, covering residues 110–544, was built on YajR; an *E. coli* proton-driven MFS antiporter crystallized in this conformation [21], with PDB code 3WDO. Primary sequence alignment of *Ca*Mdr1 and YajR (UniprotKB Q9URI1 and P7726, respectively) was performed using AlignMe [60]. The alignment was used to manually superimpose the IF model of *Ca*Mdr1 with the crystal structure of YajR with Pymol (Version 2.5.0 Schrödinger, LLC.); the superposition was then submitted to Modeller [61], which generated 20 models, from which the closer representative of the original structure was selected manually. Cytoplasmic and extracellular membrane limits were set as defined by the PPM server (https://opm.phar.umich.edu/ppm_server; accessed on 28 January 2021).

#### 2.2.9. Statistical Analyses

All plots were made using either GraphPad Prism (San Diego, CA, USA) or MS-Excel. All data are represented as mean ± SD. Statistical analyses were performed using Student’s T-test. Differences were considered statistically significant when *p* < 0.05 (* signifies *p* value ≤ 0.05, ** signifies *p* value ≤ 0.01, and *** signifies *p* value ≤ 0.001).

## 3. Results and Discussion

### 3.1. Generation of CaMdr1 Drug-Sensitive Suppressors

Using our 84 critical *Ca*Mdr1 alanine-mutant library [38], we forced yeast expressing 64 of them (see Table S2) to grow on media containing toxic concentrations of either cycloheximide (CHX), 4-nitroquinoline (4-NQO), or fluconazole (FLC) (Figure S1). Several rounds of screens selected newly drug-resistant colonies from 16 different strains (Table 1), corresponding to initial mutants that originated from most of the categories previously defined with respect to their initial impact on the antiporter [38]: *Lipids* for F216, Y408, and I448; *Structure* for G230, P257, and L480; *Mechanism* for I123, T127, and R184; and *Binding*/*Polyspecificity* for W249, Y365, Y369, F371, F474, Q478, and V506 (Figure S1). When assessing the restored drug-resistance phenotype of these strains both by the spot assay and MIC_80_ determination methods, most of the yeast-expressing mutants belonging to the *Binding* group (W249A, Y365A, Y369A_,_ F371A, and Y408A) together with I123A did not sustain their growth in the presence of drugs, indicating a transient effect which was gradually lost (Table 1, Figure S1). Sequencing the *CaMDR1* gene in the 10 other strains showed that those expressing P257A, I448A, F474A, Q478A, and V506A mutants did not have a secondary mutation inside the gene, implying an intergenic (extragenic) phenotypic effect (Table 1). Interestingly, all these residues were positioned at the interface between the inner and outer leaflets of the membrane (Figure S1). Finally, five strains displayed a secondary mutation along with the primary mutation within the *CaMDR1* gene: T127A-G140D, R184A-D235H, F216A-G260A, G230A-P528H, and L480A-A435T. All these primary alanine mutants are restricted to the *Structure*/*Lipids* (F216, G230, L480) and *Mechanism* (T127, R184) groups.

**Figure 1:**
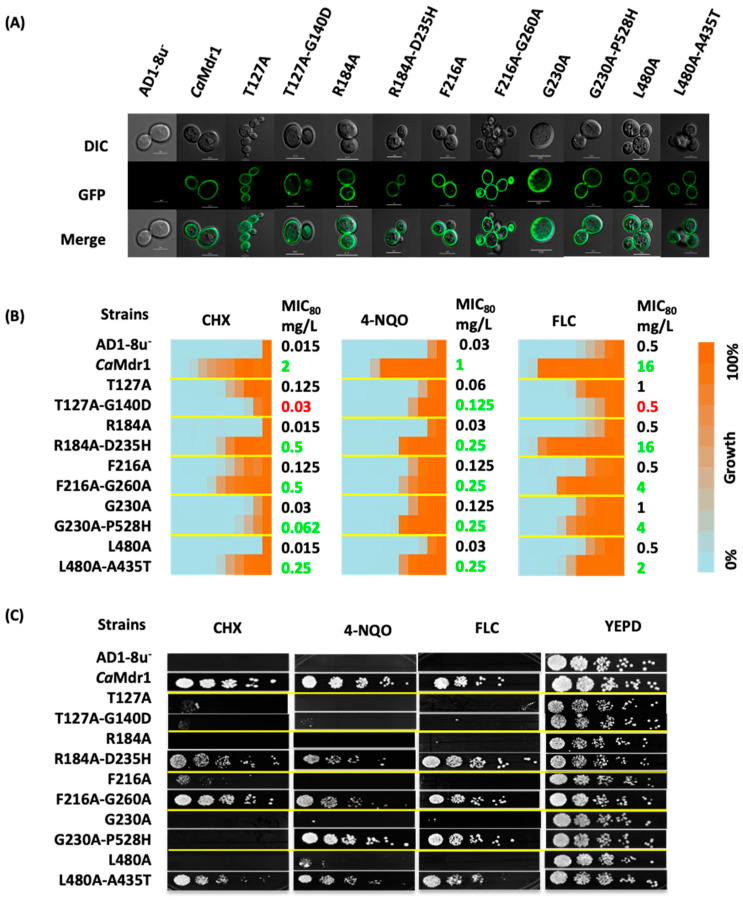
Cell localization and drug resistance profile of primary alanine mutants and their corresponding suppressor mutants. (**A**) *Ca*Mdr1 suppressor mutants localized by confocal microscopy. (**B**) Drug resistance heat map and MIC_80_ values for the corresponding strains. A 2-fold dilution was applied to 8 mg/L CHX and 4-NQO and 32 mg/L FLC. (**C**) 5-fold serial dilution spot assays of the same strains done on solid YEPD medium, with either 0.15 mg/L CHX, 0.15 mg/L 4-NQO, or 0.8 mg/L FLC added. Data were collected after 48 h of incubation at 30 °C from 3 independent experiments.

We re-constructed these five pairs of mutants in the WT *CaMDR1*-GFP gene to exclude any extragenic effects and overexpressed them in the *S. cerevisiae* AD1-8u^−^, a host strain that has proven to be an excellent heterologous system for drug transporter overexpression [56,62,63]. We designated these strains as Mdr1[L480A-A435T]-GFP, Mdr1[R184A-D235H]-GFP, Mdr1[F216A-G260A]-GFP, Mdr1[T127A-G140D]-GFP, and Mdr1[G230A-P528H]-GFP. Using confocal microscopy, GFP fluorescence confirmed the proper localization at the plasma membrane of each protein (Figure 1A).

Those strains were then subjected to drug susceptibility tests towards CHX, 4-NQO and FLC (Figure 1B,C). MIC_80_ values and spot assays showed that suppressor strains expressing the L480A-A435T, R184A-D235H, and F216A-G260A *Ca*Mdr1 variants grow in the presence of drugs with up to 30-fold MIC_80_ increase compared to their respective primary alanine mutants. Although initially isolated through resistance to CHX, the strain expressing the T127A-G140D variant hardly maintained such resistance, together with that towards FLC. However, it remained slightly more resistant to 4-NQO than the parental strain. Finally, the yeast expressing the G230A-P528H variant displayed the same sensitivity pattern to CHX as G230A variant but remained 2–4-fold more resistant to 4-NQO and FLC.

### 3.2. Most of the Drug-Sensitive Mutants and Suppressors Are Located within the Conserved Signature Motifs of DHA1 MFS

**Figure 2:**
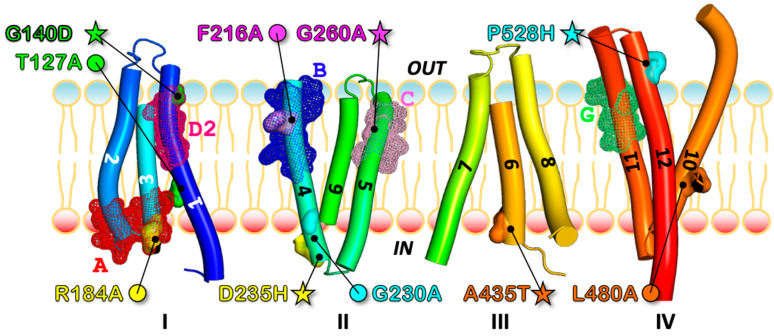
Localization of the couples of primary debilitating and secondary rescuing transport mutants of *Ca*Mdr1 with respect to MFS Signature motifs and internal structural repeats. 3D model of *Ca*Mdr1 in inward-facing conformation displayed with respect to the four structural repeats (I–IV) [28] and the conserved A, B, C, D2, and G signature motifs of the proton-dependent multidrug efflux systems [30]. See Figure S2 and Figure 3 for details. Primary debilitating (circles) and secondary rescuing (stars) mutants are shown in surface and sticks and indicated by the same color for each couple. Conserved motifs are shown in mesh form.

As evident from our 3D model, primary and secondary mutants distributed along TMH1, 3, 4, 5, 9, 10, and 12 (Figure 2), either belonging to the same TMH (1) for T127A and G140D, or (most often) to two different TMHs. In the inward-facing 3D model of *Ca*Mdr1, those pairs are either close to each other for R184A_TMH3_-D235H_TMH4_, F216A_TMH4_-G260A_TMH5_, and L480A_TMH10_-A435T_TMH9_, or far from each other for G230A_TMH4_-P528H_TMH12_ (Figure 2). Seven of the ten modified residues are in the N-domain of *Ca*Mdr1.

**Figure 3:**
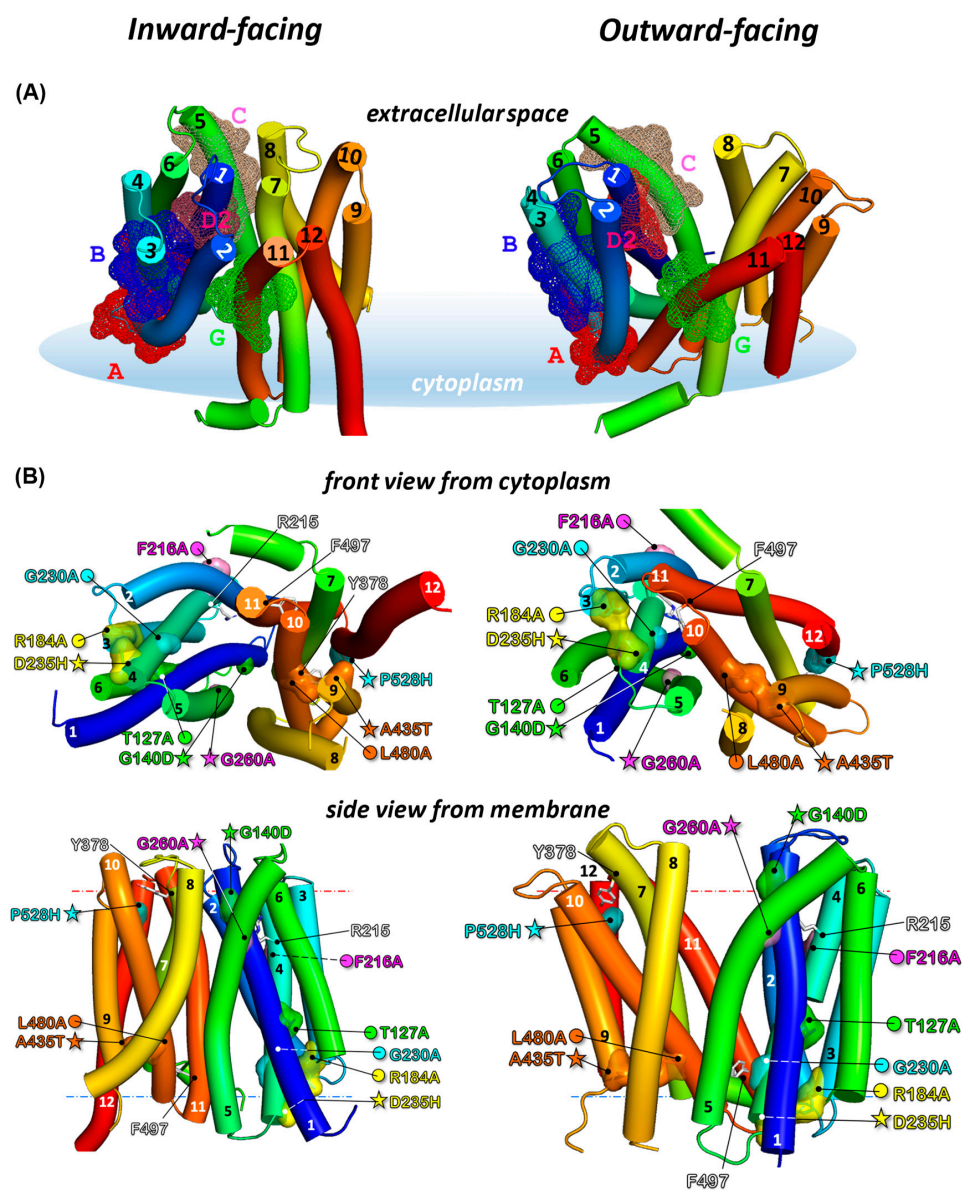
Position of conserved MFS Signature motifs and location of primary mutant and secondary suppressor couples in the inward- and outward-facing models of *Ca*Mdr1. (**A**) **Left**, inward-facing conformation based on GlpT sequence [34], optimized with Modeller. **Right**, outward-facing conformation based on YajR crystal structure [21]. Models are displayed in solid cartoon (Pymol v2.5.0), colored from the N-(blue) to the C-ends (red). Signature motifs are defined in Figure 2. (**B**) Front and side views of the inward- and outward-facing models with position of primary debilitating (circles) and secondary restoring (stars) mutants. Residues are colored by couples. Residues R215, Y378, and F497 are discussed in the text. Blue and red dotted lines indicate cytoplasmic and extracellular membrane limits as defined by the PPM server (https://opm.phar.umich.edu/ppm_server accessed on 28 January 2021).

Remarkably, looking at these mutants with respect to the DHA1 MFS subfamily motifs [30] revealed that G140 belongs to motif D2, R184 to motif A, F216 to motif B, and G260 to motif C, and that T127 and P528 are close to motifs D2 and G, respectively (Figure 2, Figure 3 and Figure S2). As introduced above, conserved residues of these motifs are thought to have a common key mechanistic role within MFS proteins, which is in line with the fact that the present restoration process was finally successful for residues of the *Mechanism* and *Structure* groups (Table 1, Figure S1).

### 3.3. Localization of Sensitive and Suppressor Mutants in the Outward-Facing Conformation of CaMdr1

To get a better view of the positional significance of mutant and suppressor pairs, we generated the 3D model of the outward-facing conformation of *Ca*Mdr1, covering residues 110–544. We built it on YajR, an *E. coli* proton-driven MFS antiporter crystallized in this conformation [21], with PDB code 3WDO. Primary sequence alignment of *Ca*Mdr1 and YajR (UniprotKB Q9URI1 and P7726, respectively) using AlignMe [60] showed 10% sequence identity and 63% matched position. The alignment was used to manually superimpose the inward-facing model of *Ca*Mdr1 with the crystal structure of YajR with Pymol. The alignment was then submitted to Modeller [61], which generated 20 models, from which the more representative was selected manually. The final outward-facing model (Figure 3A, right panel) displays a reorientation towards the external side of the membrane of the N- and C-moieties, exposing the drug-binding pocket to the extracellular space. Comparison with the inward-facing model (left panel) shows the spatial distribution of conserved signature motifs. Most of them are clustered in (A, B, C, D2) or close to (G2) the N-ter moiety. The models also highlight the remarkable alignment of B and C motifs with D2 in between. We also took advantage of the recent developments of 3D modelling offered by AlphaFold [64] to compare this model with ours, and we found it to be quite close to our inward-open model (Figure S4).

Checking the distribution of the 84 critical residues with respect to their category in this new conformation (Figure S5) showed that residues belonging to the *Binding* and *Polyspecificity* groups (green and orange, respectively) remain facing the drug-binding cavity (right panels) and those interacting with lipids (pink) are still facing them, while those involved in the mechanism (blue) remain mainly clustered in the N-moiety. In addition, residues conferring polyspecificity (orange) are still localized at the periphery of those involved in substrate binding (green), which strengthens our previous finding that such a pattern is a molecular feature of MDR pumps’ polyspecificity [38,65]. 

With these models, we positioned the different primary sensitive and secondary resistant mutant and suppressor pairs (Figure 3B). Comparing their location in our inward-open model and the AlphaFold one, we observed that the mutants and revertants are in the same place or close in both models. One main difference is the larger loop linking TMH11 and TMH12 predicted by AlphaFold, displacing P528 in that loop at the top of the protein. However, the residue remains well oriented towards Y378 (described below) and close to it (Figure S4).

### 3.4. Local Compensations Restore 3-TMHs Repeat Tandem Interactions in N- and C-Domains and Highlight the Role of Motif A

Looking first at the mutant and suppressor pairs which are spatially closer, namely R184A-D235H and L480A-A435T in the N- and C-domains, respectively (Figure 3B), a series of common features were revealed: (i) each couple is perfectly symmetrically positioned on the axis of the membrane in their respective domain; (ii) they are close to the cytoplasm side of the membrane domain; (iii) each residue belongs to a specific 3-TMH repeat—I for R184, II for D235, III for A435, and IV for L480 (Figure 2); and (iv) they remain close irrespective of whether it is the inward-facing or outward-facing conformation.

R184 brings a positive charge to the motif A where it belongs (Figure 2, Figure 3 and Figure S3) and which is engaged in a salt bridge with D235, both in inward- and outward-facing models of *Ca*Mdr1 (Figure 3 and Figure S6A). The salt bridge contributes to stabilizing the interaction between TMH3 and TMH4 and, consequently, between 3-TMH repeats I and II (Figure 2). D235 is rather well conserved (Figure S3) and indeed critical as the D235H single mutant that we generated confers full sensitivity to drugs when expressed in yeast (Figure S7A,B). This observation strengthens the hypothesis of a stabilizing role for the salt bridge, as also concluded for the corresponding residues of TetA (B), R70, and D120 [66]. However, this does not exclude a specific role of the positive charge provided by R184 since the compensation process favors the restoration of a positive charge, with the D235H substitution in the background of R184A. However, this option does not seem relevant because exploring the pH sensitivity of those mutants in the presence or absence of 4-NQO did not reveal any significant dependency on this parameter (Figure S7C,D).

In the C-domain, the L480A mutation in TMH10 is compensated by A435T in TMH9 (Figure 3), for which inward- and outward-facing models of *Ca*Mdr1 suggest that both residues interact together within a local network of hydrophobic interactions involving aliphatic (I412_TMH8_, I434_TMH9_, V437_TMH9_, I476_TMH10_, M484_TMH10_) and aromatic (Y408_TMH8_, F477_TMH10_, F481_TMH10_) residues (Figure S6B). Therefore, the size reduction of the aliphatic tail of L480 to alanine and the central location of the couple of residues seems sufficient to weaken such a network. This hypothesis is strengthened by the effect of the A435G mutation that we found previously to be fully deleterious for multi-drug efflux [38], in contrast to the single A435T mutation that we generated (Figure S6C). Introducing the two mutations L480A and A435T in the inward- and outward-facing models of *Ca*Mdr1 shows that T435 may be well positioned to generate a H-bond with the Sulphur atom of M484, one helix turn below L480 on TMH10 (Figure S6B), which may contribute to restoring the interaction lost with the L480A mutation.

Altogether, these data suggest that the regions in which the two pairs of mutants and suppressors are located grant a stable interaction between their respective TMHs, allowing synchronization of the corresponding pairs of 3-TMH repeats in each domain along the drug translocation process. The well-conserved motif A might indeed play this role in MFS proteins. This could also be the case for the regions encompassing A435 and L480, although with a lower conservation level compensated by an enrichment in aliphatic and aromatic residues (Figure S3). The natural abundance and variability of such residues reduce the requirement of identity since hydrophobic properties are preserved, which therefore masks a potential signature. 

### 3.5. Distant Compensations in the N-Domain Highlight Interplay between Motifs A, B, and D2 

Looking at the mutant and suppressor pair F216A-G260A revealed that both residues belong to the same 3-TMH repeat II and that they are part of motif B in TMH4 and motif C in TMH5, respectively (Figure 2). F216 and G260 stand in the same plane in the extracellular leaflet of the membrane, at a distance of ~20 Å from each other, both in inward- and outward-facing models (Figure 3B). F216 is rather well conserved in motif B (Figure S3), together with F220 positioned one helix turn below, which we also previously identified as critical when mutated into alanine [38]. Inward- and outward-facing models of *Ca*Mdr1 show that both aromatic residues face lipids (Figure 3B), suggesting that their replacement by an alanine might increase the level of freedom of the corresponding segment of TMH4 in the outer leaflet. This may alter the precise location of R215 that follows F216 on the opposite face of TMH6 and points to TMH1 in the center of the membrane domain (Figure 3), where it plays the main role in proton antiporting [34]. In the background of the F216A mutation, drug efflux is restored through the G260A substitution in TMH5, pointing at the same level as R215 to the other side of TMH1 (Figure 3). As a member of the motif C, G260 is the central glycine residue of a double glycine motif G^256^-X-X-X-G^260^-X-X-X-G^264^ (Figure S3), which is typical of membrane helix–helix interaction [35] and faces the outer leaflet part of TMH1. The glycine motif contributes to the tight and constant interaction between TMH4 and TMH1. Glycine substitution to alanine is possible in such a motif; indeed, we previously found that such a substitution is not deleterious [38]. However, a methyl group, which is bulkier than a proton, would probably push the outward leaflet part of TMH1 towards TMH5, which would contribute to reconnecting R215 to the proton translocation network.

The region of TMH1 to which R215 and G260 point to corresponds to the motif D2 (Figure 2 and Figure 3), which we found here to be targeted by the genetics strategy, with G140D restoring the drug resistance lost with the T127A mutation. T127 precedes the motif D2 while G140 is located at its C-ter end (Figure S3). G140 faces G260 in the models of *Ca*Mdr1, and its substitution by an aspartic residue may produce the same repositioning effect as the G260A mutation but also contribute to reconnecting the proton translocation network. 

Altogether, these data show that motifs B, C, and D2 constitute a bundle finely adjusted to synchronize the motion of TMH1, 4, and 5 for granting proton translocation during drug efflux. 

### 3.6. Long-Range Compensation between the N- and C-Domains

**Figure 4:**
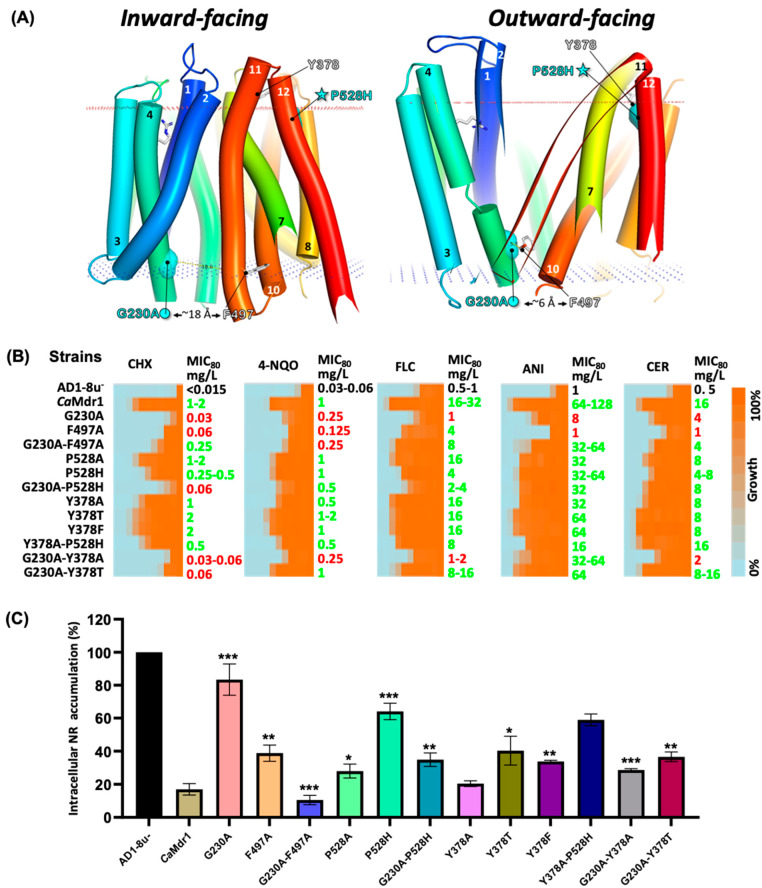
Short– and long–range interactions involving positions 230, 378, 497, and 528. (**A**). Cartoon representation of inward- and outward-facing models of *Ca*Mdr1 with membrane limits. Settings are as in Figure 3. TMH7, 11, and 12 have been partially masked. (**B**). MIC_80_ values as described in Figure 1. Anisomycin (ANI, 10 mg/L) and cerulenin (CER, 4 mg/L) have been added to the screen. (**C**). Nile red (NR) accumulation assays in host AD1-8u-, WT *Ca*Mdr1-GFP, and variants. NR accumulation in host strain was set to 100%. Results are the mean of three independent cultures. All single mutants were compared with WT strain while double mutants were compared with their corresponding primary single mutants. Differences were considered statistically significant when *p* < 0.05 (* signifies *p* value ≤ 0.05, ** signifies *p* value ≤ 0.01, and *** signifies *p* value ≤ 0.001).

The pair G230A-P528H constitutes a striking event of long-range compensation. G230 is located in the C-end of TMH4 within the 3-TMH repeat II of the N-domain, close to the cytoplasmic side of the protein and oriented towards the drug-translocation pathway. P528 is symmetrically located about 40 Å away at the external face of the membrane in the N-end of TMH12 in the 3-TMH repeat IV of the C-domain (Figure 2, Figure 3B and Figure 4). TMH11 seems to be the most direct link between the couple of residues (Figure 4) and, interestingly, carries the G motif in the outer leaflet region of the TMH (Figure 2). Inward-open and outward-open models of *Ca*Mdr1 suggest that TMH11 undergoes a large movement by which its N-Ter comes close to G230 in the outward-open conformation (Figure 4A). Here, residue F497 in TMH11 may be as close as G230 thanks to the empty space provided by the absence of the lateral chain of G230, as in a glycine motif. The distance increases up to about 20 Å when coming back to the inward-facing state (Figure 4). This interaction may therefore contribute to the stabilization of the outward-facing state, weakened or hampered by the G230A substitution that locally increases the steric hindrance.

According to this scenario, reducing the steric hindrance at position 497 should have a compensatory effect, which we evaluated by generating and studying the effect of the F497A mutation, alone and in the background of G230A (Figure 4B and Figure S8). Both variants were well expressed and localized at the cell surface in yeast (Figure S8A). Liquid (Figure 4B) and solid (Figure S8B) dilution assays showed that the yeast expressing the F497A variant, except for fluconazole, becomes sensitive to cycloheximide and 4-nitroquinoline, and even more so to anisomycin and cerulenin. These results confirm that reducing the steric hindrance at position 497 has the same deleterious impact as increasing it at position 230. As expected, the strain expressing the *Ca*Mdr1 variant F497A in the background of G230A recovers a significant level of resistance towards most of the antifungals (Figure 4B) and full resistance towards Nile red (Figure 4C). These data thus support the functional proximity and steric complementarity of G230 and F497 suggested by the 3D models, together with, unexpectedly, a role of these residues and their interaction in drug selectivity.

Exploring the P528 region with the same strategy (Figure 4B and Figure S8), with the addition of accumulation assays carried out with Nile red (Figure 4C), showed that the yeast expressing the *Ca*Mdr1 P528H variant remains significantly resistant to all drugs except Nile red, indicating a limited impact of this mutation when present alone. Replacement by an alanine gave the same result. However, introducing the P528H mutation in the background of the deleterious G230A mutation restores a better level of resistance towards the tested drugs, and accumulated significantly lower amounts of Nile red compared to the yeast expressing the single G230A variant.

Inward- and outward-open models of *Ca*Mdr1 suggest that P528 faces TMH7, 8, and 11 (Figure 4 and Figure S9). A histidine residue at this position might locally increase the steric hindrance but also bring polarity and charge, which together probably contribute to repositioning TMH12 with respect to the other TMHs for compensating for the handicap introduced with G230A. We noticed that in both models, P528 is particularly close (~10 Å) (considering Cα, as also in the AlphaFold model; Figure S4) to Y378 in TMH7 (Figure 3, Figure 4 and Figure S9), which, interestingly, stands in the well-conserved short segment P*h*Y*h* (*h* for hydrophobic) (Figure S3). To gain further insights into the local and distant region interplay, we generated a series of single and double mutants and analyzed their impact on *Ca*Mdr1 substrate efflux (Figure 4 and Figure S8). Substituting Y378 by an alanine, threonine, or phenylalanine did not produce any significant effect on substrate efflux, even in the background of P528H. Addition of the secondary P528H mutation in the background of Y378A was more deleterious. However, an alanine or threonine at position 378 in the background of G230A restored a resistance phenotype, confirming that P528 and Y378 are indeed close. Altogether, these data show that TMH11 tunes the relative positions of the N- and C-domains, mainly for allowing the inward- to outward-open conformational change, but with consequences for drug selectivity. 

## 4. Conclusions

In this study, we describe how a non-directed mutagenesis process applied to an MDR drug:H^+^ antiporter selects primary and secondary mutants mainly located in a few conserved stretches of proteins belonging to the DHA1 MFS family, the so-called signature motifs A, B, C, D2, and G. Despite the functional diversity of the initial 64-mutant library, only mutants initially having a deleterious impact either on the mechanism (corresponding to residues with small or bulkier lateral chain pointing inside the protein but not in the drug-binding cavity [38]) or the interaction with lipids (residues with lateral chain facing the membrane [38]) allow a rescuing secondary mutation to occur. Therefore, the privileged distribution of these residues among the signature motifs suggests that these regions may constitute the mechanistic core of DHA1 MFS proteins. Clustered in the outward leaflet, motifs B, C, and D2 may provide the power stroke of the efflux, while motifs A and G, symmetrically located in the inner and outer leaflet of the membrane, may drive the conformational change allowing drug translocation.

## Figures and Tables

**Table 1:** Position, location, and drug profiling of alanine mutants and suppressors. Phenotypes of alanine mutants and suppressor colonies are classified as TS (Total Susceptibility), SS (Selective Susceptibility), TR (Total Resistance), SR (Selective Resistance), or PR (Partial Resistance).

	Primary Mutant				Suppressor	
Categories	Mutation	Location	Phenotype	Function (Redhu et al., 2018)	Drug Leading to Resistance	Phenotype
Transiently resistant strains	I123A	TMH-1	SS	drug/H^+^ antiport mechanism	CHX, 4-NQO	TS
	W249A	TMH-5	TS	drug binding and transport	CHX	TS
	Y365A	TMH-7	TS	drug binding and transport	CHX, 4-NQO	TS
	Y369A	TMH-7	TS	drug binding and transport	CHX	PR
	F371A	TMH-7	TS	drug binding and transport	CHX, 4-NQO	PR
	Y408A	TMH-8	TS	exposed to lipid interface	4-NQO	PR
Resistance phenotype (intergenic mutation)	P257A	TMH-5	TS	structural integrity	CHX	TR
	I448A	TMH-9	TS	Crucial for plasma membrane localization	CHX, 4-NQO	TR
	F474A	TMH-10	TS	drug binding and transport	CHX	SR
	Q478A	TMH-10	TS	drug binding and transport	CHX	SR
	V506A	TMH-11	TS	Crucial role in polyspecific substrate binding and transport	CHX	TR
Resistance phenotype (intragenic mutation)	T127A	TMH-1	TS	drug/H^+^ antiport mechanism	CHX	SR
	R184A	TMH-3	TS	drug/H^+^ antiport mechanism	CHX	TR
	F216A	TMH-4	TS	exposed to lipid interface	CHX	TR
	G230A	TMH-4	TS	structural integrity	CHX	SR
	L480A	TMH-10	TS	structural integrity	4-NQO	TR

## Data Availability

Not applicable.

## Supplementary Materials

The following supporting information can be downloaded at https://www.mdpi.com/article/10.3390/jof8050538/s1. Figure S1: Strategy of drug-resistant strain isolation and mapping of residues screened; Figure S2: *Candida albicans* Multidrug resistance protein 1 (Uniprot # Q9URI1) primary sequence and localization of conserved motifs and mutants of the study; Figure S3: Weblogo representation of DHA1 subfamily of MFS transporters; Figure S4: Location of critical residues in the Alphafold 3D model of *Ca*Mdr1; Figure S5: Location of critical residues in the 3D models of *Ca*Mdr1 in inward- and outward-facing conformations; Figure S6: Location details of mutant and suppressor couples R184A-D235H and L480A-A435T and spot dilution assay of the A435T single mutant; Figure S7: Exploration by spot dilution assays of the pH dependency of single and double mutants of the R184-D235 couple; Figure: S8: Phenotypic characterization of yeast strains expressing single secondary mutants *Ca*Mdr1; Figure S9: Relative position of P/H528 and Y378 in the inward- and outward-facing models of *Ca*Mdr1; Table S1: Oligonucleotides used in the study; Table S2: Yeast strains used and generated in this study.

## Abbreviations

MFS—Major facilitator superfamily, *Ca*Mdr1*—Candida albicans* Multidrug resistance 1 protein, DHA-1—Drug:H^+^ antiporter-1 subfamily; TMD—Transmembrane domain; TMS—Transmembrane segment; TMH—Transmembrane helix, ECL—Extracellular loop; ICL—Intracellular loop; CCL—Central cytoplasmic loop; CHX—Cycloheximide; 4-NQO—4-Nitroquinoline; FLC—Fluconazole; ANI—Anisomycin; CER—Cerulenin; NR—Nile red; DMSO—Dimethyl sulfoxide; WT—Wild type; PM—Plasma membrane, ABC—ATP binding cassette, SDM—Site-directed mutagenesis.
