# Supplementary material for: Spontaneous Suppressors against Debilitating Transmembrane Mutants of CaMdr1 Disclose Novel Interdomain Communication via Signature Motifs of the Major Facilitator Superfamily
Source: J Fungi (Basel). 2022 May 22;8(5):538. doi: 10.3390/jof8050538 (PMC9143388; doi:10.3390/jof8050538)
Supplement: Supplementary file 1 [file jof-08-00538-s001.zip › jof-1711943-supplementary.pdf]

Supplementary Materials

Table S1. Oligonucleotides used in the study.

Primer Name	Primer Sequence
CaMdr1/F	GCATTACAGATTTTTGAGAGATAGTTTTGTTGG
CaMdr1/R	GCATACTTAGATCTTGCTCTCAATTTTGGTCC
CaMdr1 T127A/F	CAAATTTCATTTTTGGCAACTTCAGTTTATATGG
CaMdr1 T127A/R	CCATATAAACTGAAGTTGCCAAAAATGAAATTTG
CaMdr1 G140D/F	GCAGTTTATACCCCTGATATTGAAGAATTAATGC
CaMdr1 G140D/R	GCATTAATTCTTCAATATCAGGGGTATAAACTGC
CaMdr1 R184A/F	GCTATATTTGGTGCTACATCCATATATATC
CaMdr1 R184A/R	GATATATATGGATGTAGCACCAAATATAGC
CaMdr1 D235H/F	GCAAGTGTTGCTCATGTGGTTAAATTTTGG
CaMdr1 D235H/R	CCAAAATTTAACCACATGAGCAACACTTGC
CaMdr1 F216A/F	GTATATTGAGAGCCTTGGGTGGATTC
CaMdr1 F216A/R	GAATCCACCCAAGGCTCTCAATATAC
CaMdr1 G230A/F	GGCTACTGGTGCTGCAAGTGTTGCTGATGTGG
CaMdr1 G230A/R	CCACATCAGCAACACTTGCAGCACCAGTAGCC
CaMdr1 G260A/F	GTGGTCCTAGTTTTGCTCCATTCTTTGGTTC
CaMdr1 G260A/R	GAACCAAAGAATGGAGCAAAACTAGGACCAC
CaMdr1 Y378F/F	CGAAGTTTTCCCAATTTTCTTCGTTGGAGTTAAAC
CaMdr1 Y378F/R	GTTTAACTCCAACGAAGAAAATTGGGAAAACTTCG
CaMdr1 Y378A/F	CGAAGTTTTCCCAATTGCTTTCGTTGGAGTTAAAC
CaMdr1 Y378A/R	GTTTAACTCCAACGAAAGCAATTGGGAAAACTTCG
CaMdr1 Y378T/F	CGAAGTTTTCCCAATTACTTTCGTTGGAGTTAAAC
CaMdr1 Y378T/R	GTTTAACTCCAACGAAAGTAATTGGGAAAACTTCG
CaMdr1 A435T/F	GTGTTTATTCCAATTACCATTGTTGGTGGTATC
CaMdr1 A435T/R	GATACCACCAACAATGGTAATTGGAATAAACAC
CaMdr1 L480A/F	GATTTTCCAAACAGCATTCAATTTCATGGG
CaMdr1 L480A/R	CCCATGAAATTGAATGCTGTTTGGAAAATC
CaMdr1 F497A/F	TATATTGCTTCA GTTGCTGCATCAAATGATTTG
CaMdr1 F497A/R	CAAATCATTTGATGCAGCAACTGAAGCAATATA
CaMdr1 P528H/F	GGCTACCCCTGAATATCATGTTGCTTGGGGTAG
CaMdr1 P528H/R	CTACCCCAAGCAACATGATATTCAGGGGTAGCC
CaMdr1 P528A/F	GGCTACCCCTGAATATGCAGTTGCTTGGGG
CaMdr1 P528A/R	CCCCAAGCAACTGCATATTCAGGGGTAGCC

Table S2. Yeast strains used and generated in this study.

Strains	Genotype or description	Source
AD1-8Ura-	(Mata,pdr1-3,ura3 his1, Δyor1::hisG, Δsnq2::hisG, Δpdr5::hisG,Δpdr10::hisG, Δpdr11::hisG, Δycf1::hisG, Δpdr3::hisG, Δpdr15::hisG)	Kenjirou et al. 2001 [63]
AD-MDR1-GFP	AD1-8u- cells harboring MDR1-GFP ORF integrated at PDR5 locus	Ritu et al. 2007 [37]

AD-MDR1 cells carrying the following mutation in MDR1-GFP ORF and integrated at PDR5 locus Redhu et al. 2018 [38]

TMH1: I123A, T127A, T128A, S129A, Y131A, M132A, D147A

TMH2: L161A, F162A, V163A, Y166A, G167A, R184A

TMH3: Y188A, T191A, 195A, Q199A

TMH4: R215A, F216A, F220A, S223A, P224A, T228A, G229A, G230A

TMH5: W249A; P257A

TMH6: I283A, T294A, L295A

TMH7: V353A, Y360A, I361A, V364A, Y365A, L368A, Y369A, L370A, F371A, F372A
TMH8: Y394A, V398A, I399A, F406A, Y408A, P410A, E429A
TMH9: G438A, G439A, I448A,
TMHS10: A466G, 470G, F474A, I476A, F477A, Q478A, L480A
TMH11: V495A, N500A, R504A, S509A
**AD-MDR1 cells carrying the following mutations in MDR1-GFP ORF and integrated at PDR5 locus. This study**
T127A, G140D, T127A-G140D, R184A, D235H, R184A-D235H, F216A, G260A, F216A-G260A, A435T, L480A, L480A-A435T, G230A, P528H, G230A-P528H, Y378A, Y378T, Y378F, G230A-Y378A, G230A-Y378T, Y378A-P528H, P528A, F497A, G230A-F497A

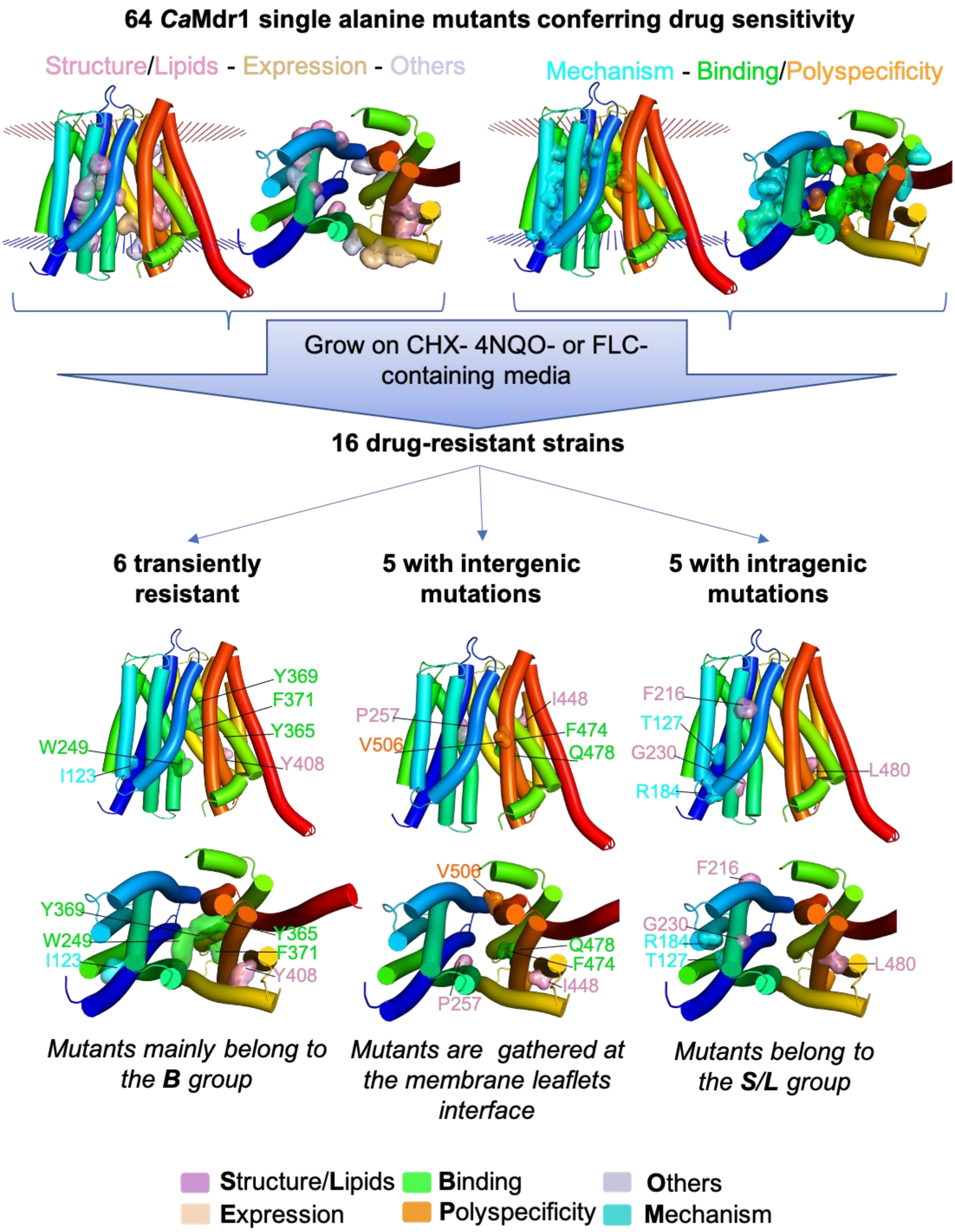

Figure S1. Strategy of drug-resistant strain isolation and mapping of residues screened. GlpT-based 3D model of *Ca*Mdr1 in inward-facing conformation (Redhu et al. 2016 [34]) optimized with Modeller in this study. TMHs limits are defined with the OPM server (https://opm.phar.umich.edu/ppm_server). The 3D model is shown in filled cylinders colored in rainbow from the N-terminus (blue) to the C-terminus (red). Screened residues are shown in surface and colored in respect of their group as indicated and previously defined Redhu et al. 2018 [38].

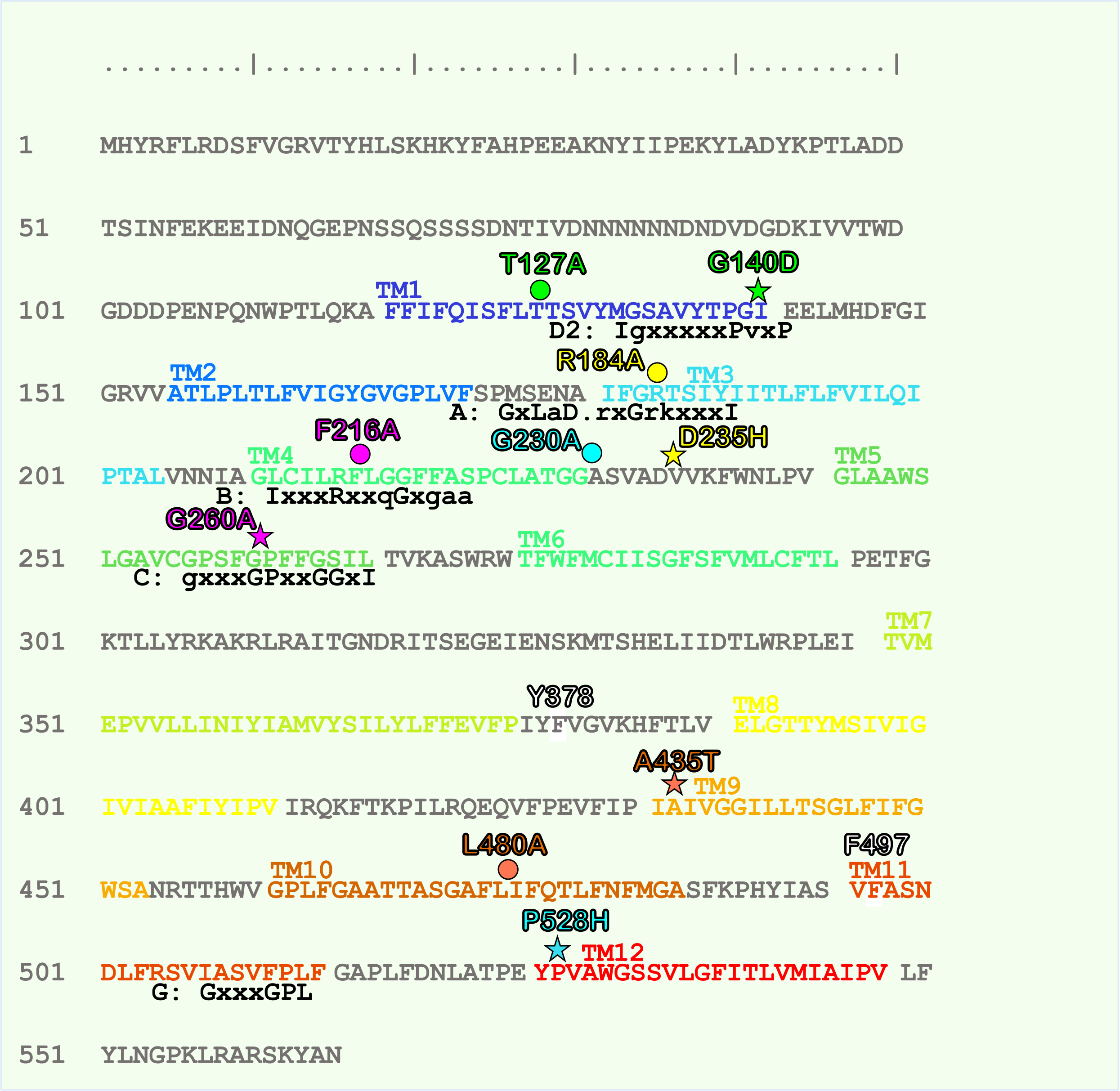

Figure S2. ***Candida albicans*** **Multidrug resistance protein 1 (Uniprot # Q9URI1) primary sequence and localization of conserved motifs and mutants of the study.** TMHs are rainbow-colored in respect of the GlpT-based 3D model (Redhu et al. 2016 [34]) optimized here. TMHs limits are defined with the OPM server (https://opm.phar.umich.edu/ppm_server). Each couple of primary-debilitating (circle) and secondary-rescuing (star) transport mutants are colored as in Figure 1. Signature motifs of proton-dependent multidrug efflux systems are defined as in Paulsen et al. 1996 [30] (see Figure S3).

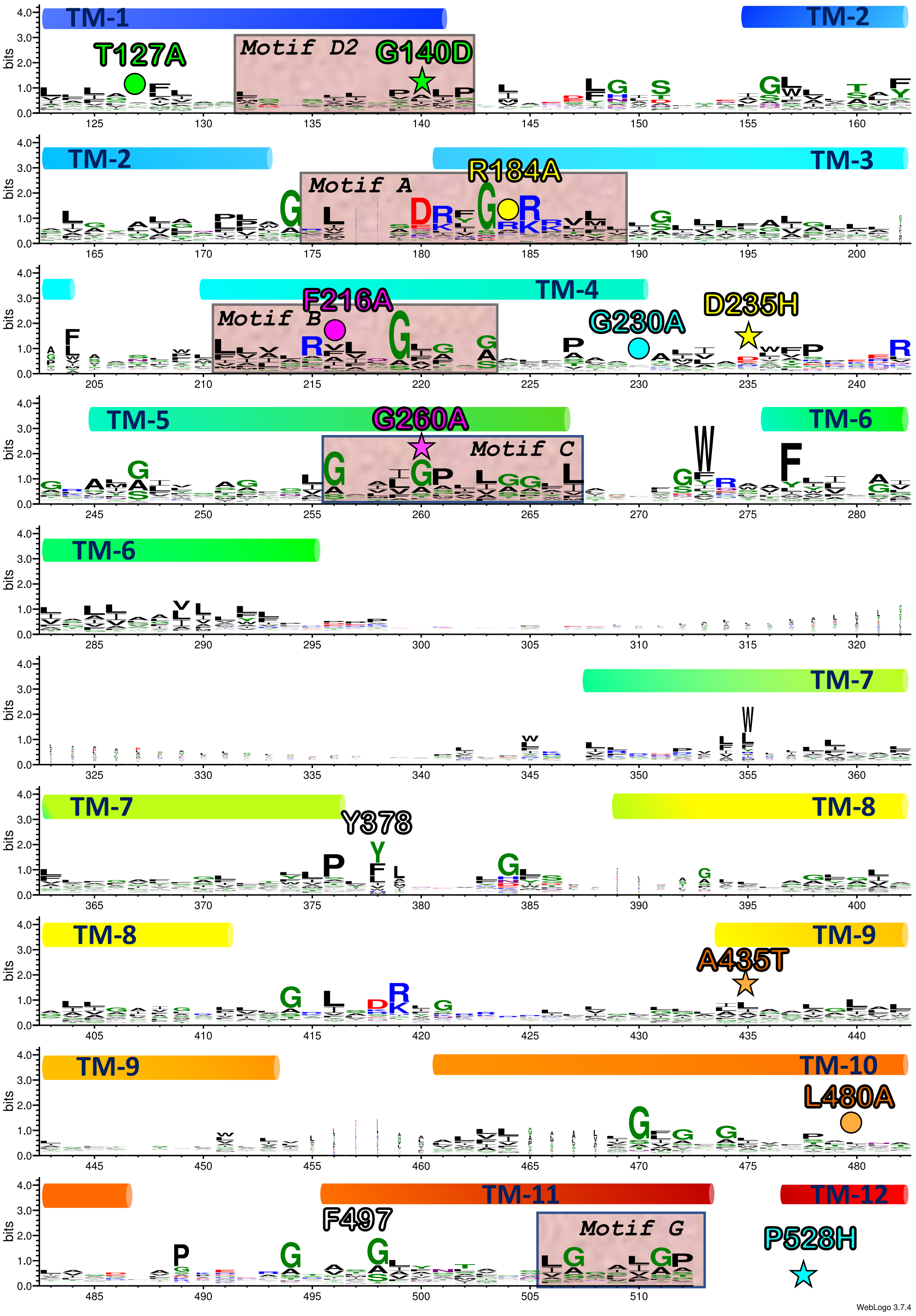

Figure S3. Weblogo representation of DHA1 subfamily of MFS transporters. Sequences are from the PFAM web server using 192 "seed" sequences from the MFS_DHA1 subfamily (PF07690). Sequence alignment is done with Jalview (2.11.1.4). Weblogo is generated by WebLogo 3. Residues are colored using the Chemistry color code: polar-green; neutral-purple; basic-blue; acidic-red; hydrophobic-black. X-axis denotes amino acid residue number of *Ca*Mdr1.

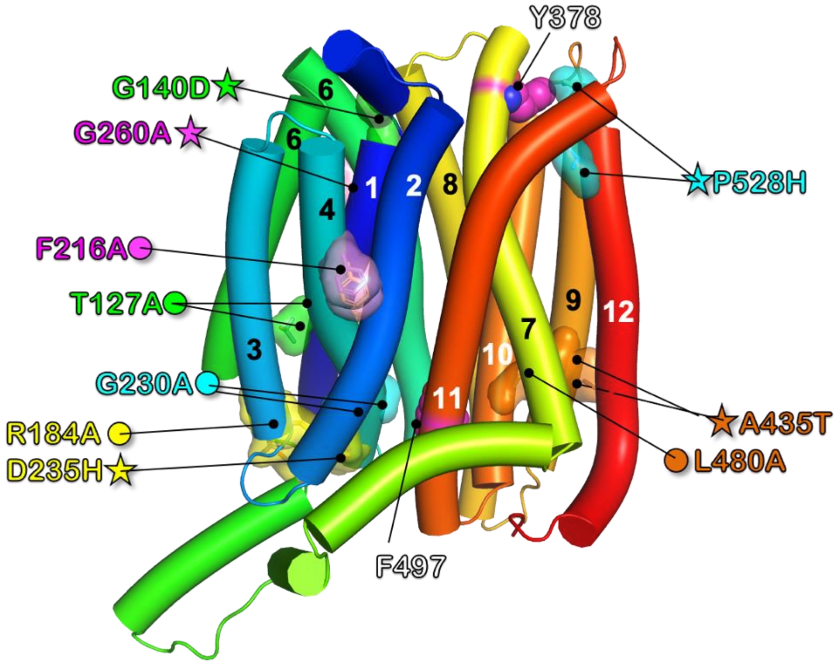

Figure S4. Location of critical residues in the AlphaFold 3D model of *Ca*Mdr1. 3D model of *Ca*Mdr1 from the AlphaFold database (AF-Q5ABU7-F1-model_v1) using the UniProt entry number Q5ABU7. The picture only shows the TMH region of the model in which pairs of debilitating and rescuing mutations are displayed as in Figure 2. Residues Y378 and F497 described later are also shown.

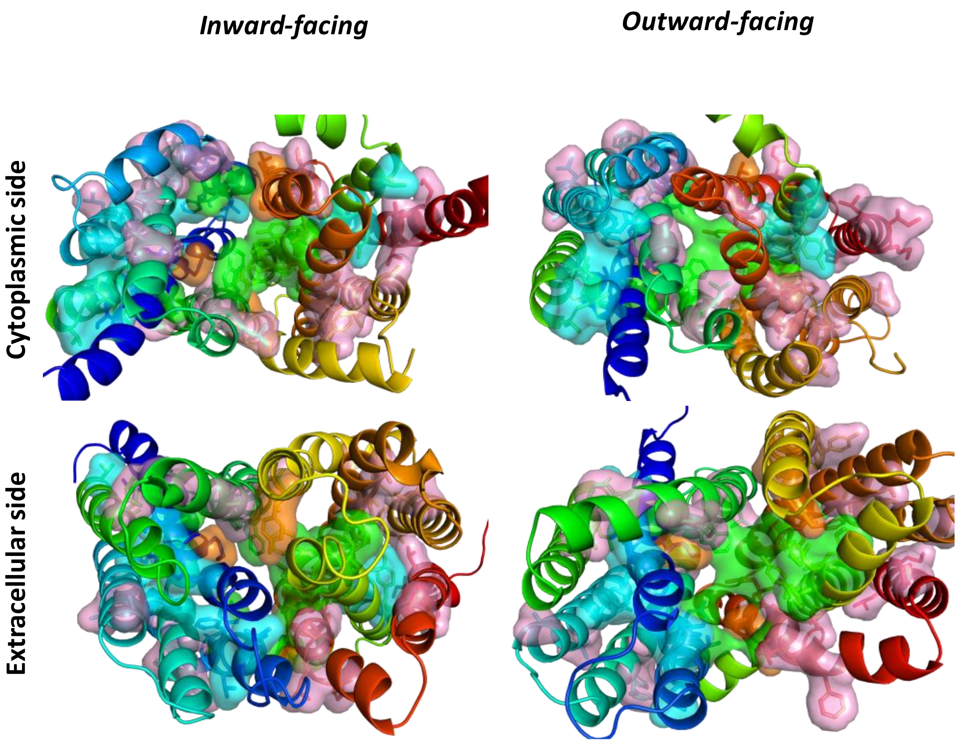

Figure S5. Location of critical residues in the 3D models of *Ca*Mdr1 in inward- and outward-facing conformations. Critical residues from the alanine mutants library (Redhu et al. 2018 [38]) are shown in surface and stick modes and colored in respect of their impact on either the mechanism (blue), interaction with lipids and structure (pink), ligand binding (green) and polyspecificity (orange)

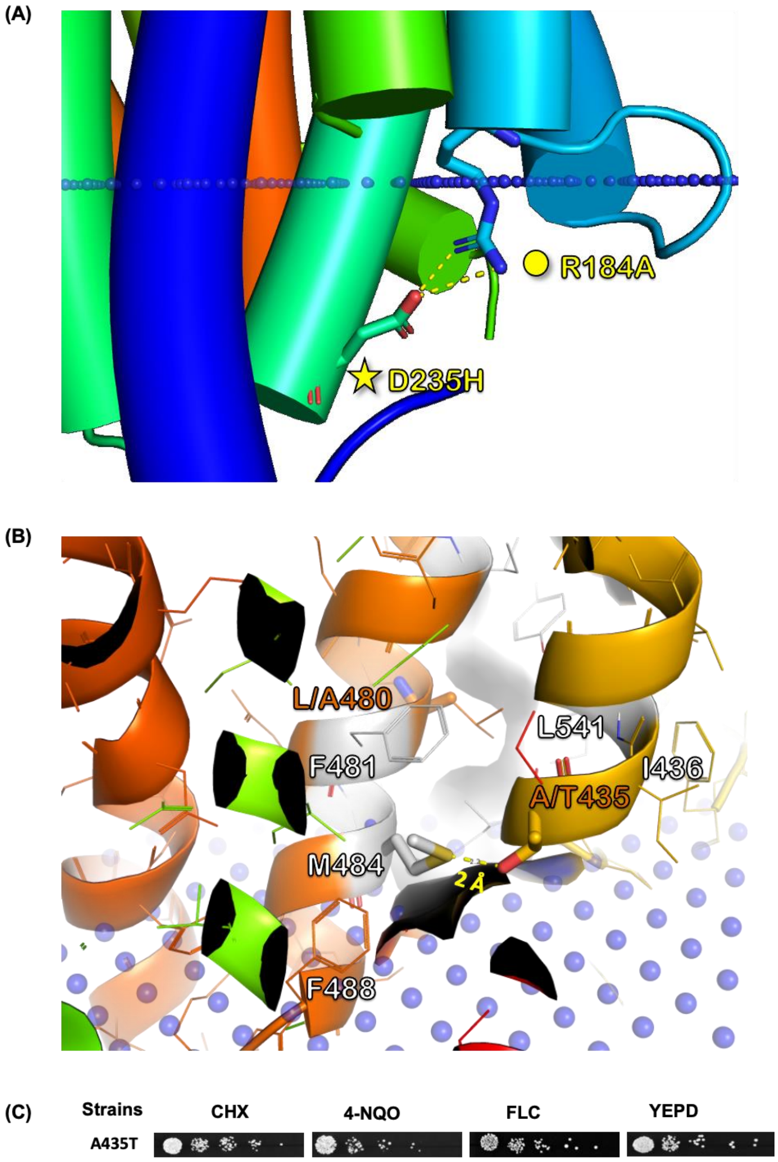

Figure S6. Location details of mutant and suppressor couples R184A-D235H and L480A-A435T and spot dilution assay of the A435T single mutant. Residues are rainbow-coloured from the N- (blue) to the C-ter (red). Blue dots indicate cytoplasmic membrane limits as defined by the PPM server (https://opm.phar.umich.edu/ppm_server). (A) Details of the salt bridge between R184 and D235 in *Ca*Mdr1 WT (B) Zoom in showing the replacement of each residue and polar interaction of the OH of T435 with the sulphur atom of M484. (C) spot dilution assay of the A435T single mutant.

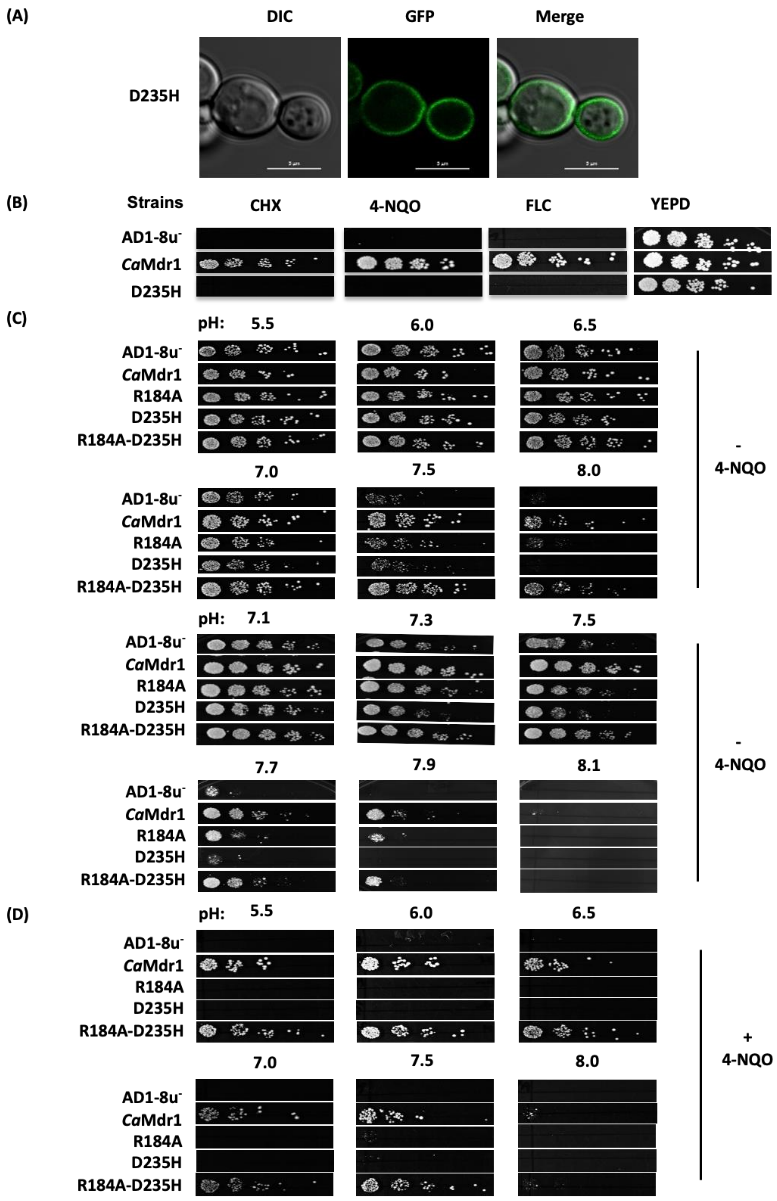

Figure S7. Exploration by spot dilution assays of the pH dependency of single and double mutants of the R184-D235 couple. (**A**) Localization and (**B**) drug sensitivity of the D235H mutant. (**C,D**) pH dependency in respect of pH, with or without drug as indicated.

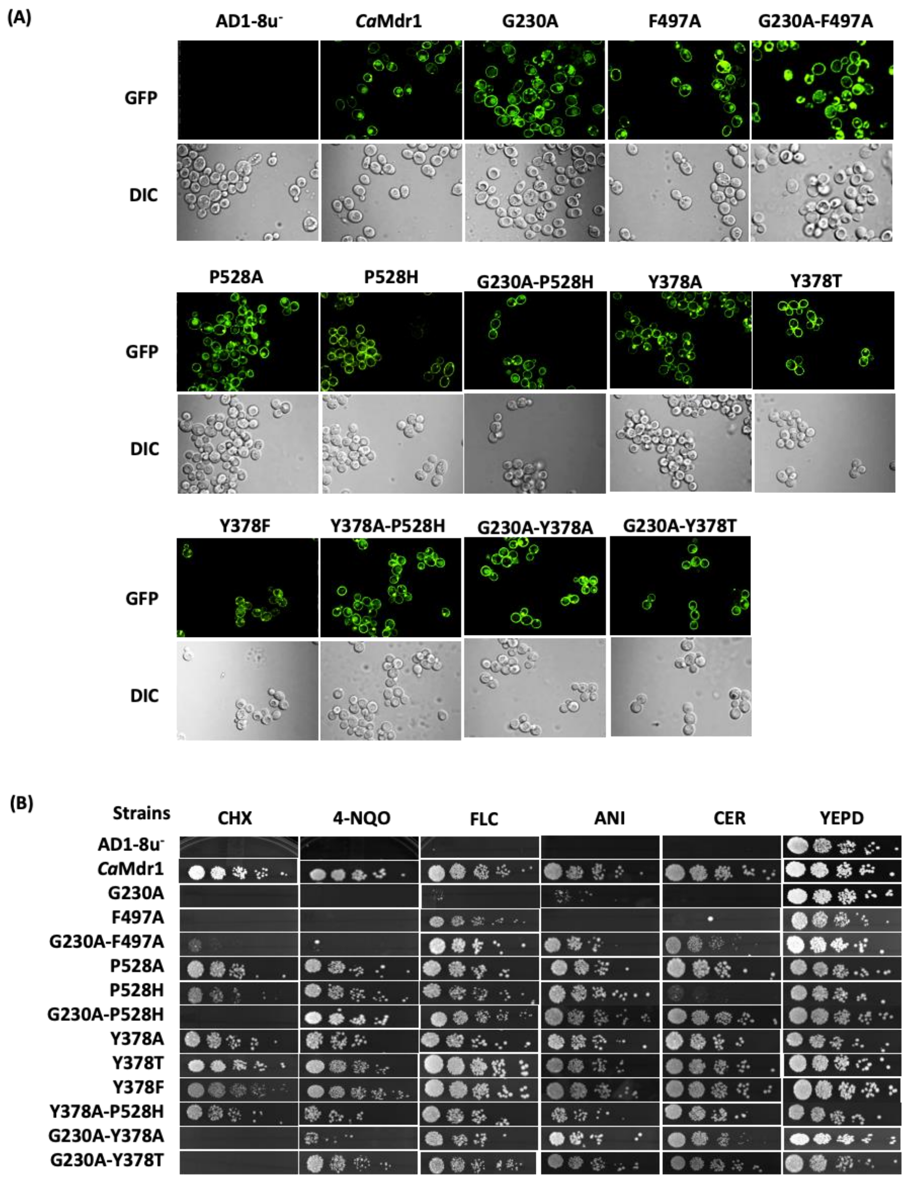

Figure S8. Phenotypic characterization of yeast strains expressing single secondary mutants of *Ca*Mdr1. **(A)** Fluorescence imaging by confocal microscope showing PM localization of AD1-8u- (control), *Ca*Mdr1-GFP (WT), mutant and reconstructed suppressor with corresponding differential interference contrast (DIC) images and merged images**. (B)** Comparison of growth by spot dilution assays for mutant variants on different *Ca*Mdr1 substrates as CHX (0.1 mg/L), 4- NQO (0.15 mg/L), FLC (0.8 mg/L), ANI (10 mg/L) and CER (4 mg/L) in YEPD agar plates. Images were captured after 48 hours of incubation at 30 °C.

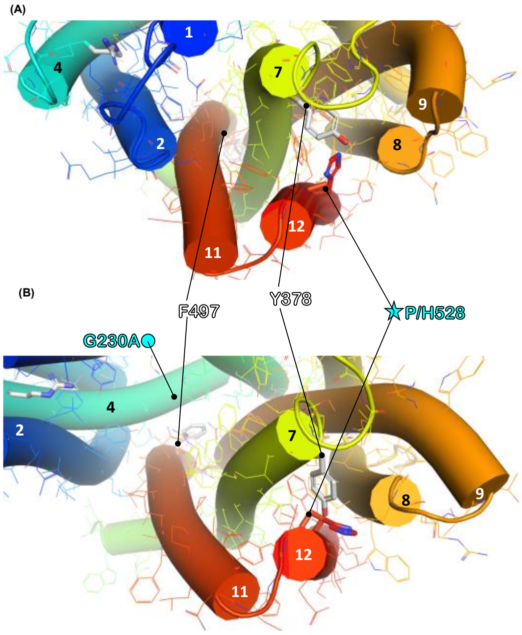

Figure S9. Relative position of P/H528 and Y378 in the inward- and outward-facing models of *Ca*Mdr1. **(A)** view from the extracellular side in inward- and (**B**) outward- facing conformations. Structural settings are as in Figure 2.
